# The Diagnosis and Management of Cutaneous Metastases from Melanoma

**DOI:** 10.3390/ijms241914535

**Published:** 2023-09-26

**Authors:** Cosimo Di Raimondo, Flavia Lozzi, Pier Paolo Di Domenico, Elena Campione, Luca Bianchi

**Affiliations:** Department of Dermatology, University of Roma Tor Vergata, 00133 Rome, Italy; flavia.lozzi@hotmail.com (F.L.); pierpaolo2727@gmail.com (P.P.D.D.); campioneelena@hotmail.com (E.C.); luca.bianchi@uniroma2.it (L.B.)

**Keywords:** melanoma, cutaneous metastases of melanoma, immunotherapy, skin cancer

## Abstract

Melanoma is one of the deadliest skin tumors, accounting for almost 90% of skin cancer mortality. Although immune therapy and targeted therapy have dramatically changed the prognosis of metastatic melanoma, many patients experience disease progression despite the currently available new treatments. Skin metastases from melanoma represent a relatively common event as first sign of advanced disease or a sign of recurrence. Skin metastases are usually asymptomatic, although in advanced stages, they can present with ulceration, bleeding, and superinfection; furthermore, they can cause symptoms related to compression on nearby tissues. Treatments vary from simple surgery resections to topical or intralesional local injections, or a combination of these techniques with the most recent systemic immune or target therapies. New research and studies should focus on the pathogenesis and molecular mechanisms of the cutaneous metastases of melanoma in order to shed light on the mechanisms underlying the different behavior and prognoses of different patients.

## 1. Introduction

Melanoma is one of the deadliest skin tumors, accounting for almost 90% of skin cancer mortality [1]. In the last decade, the incidence of melanoma has significantly increased, especially in patients over the age of 60, although rising rates of incidence have also been registered in young and middle-aged patients [2]. The main cause of death related to melanoma is metastatic spread to the viscera, with the lymph nodes being the first and the lungs being the most common site of metastases [3].

Although immune therapy and targeted therapy have dramatically changed the prognosis of metastatic melanoma, many patients experience disease progression despite the currently available new treatments [4]. Therefore, metastatic melanoma still remains a challenging disease with a need for new combination treatments. Currently, pivotal randomized clinical trials are focusing on promising combinatorial approaches for patients with unresectable stage III or IV melanoma that have experienced primary and secondary resistance to immune-checkpoint blockers or BRAF and MEK inhibitors [5,6].

On this matter, properly identifying and treating cutaneous metastases from melanoma (CMM) can be challenging due to the lack of effective treatments, thus having a deep impact on patients’ quality of life.

Herein, we will review the clinical and therapeutic aspects of CMM.

## 2. Classification

Skin metastases from melanoma represent a relatively common event as the first sign of advanced disease or a sign of recurrence [7]. While breast and colon cancer have the highest rates of metastasizing to the skin [8], the incidence of CMM is constantly increasing, accounting for 10–17% of patients affected by melanoma [7]. In 2–8% of patients, skin metastases represent the first manifestation of melanoma [9]. According to the distance from the primary melanoma, skin metastases can be classified as satellite metastases (defined as up to 2 cm from the primary tumor), in-transit metastases (ITM) (localized in the skin between 2 cm from the site of the primary tumor and the first draining lymph node), and distant metastases (defined as lesions that arise in any skin site) [10]. According to the eighth American Joint Committee on Cancer (AJCC) melanoma staging system, both satellite and ITM define a stage III disease, while any distant metastasis, with or without visceral metastases, identifies a stage IV disease [10]. Patients that have metastases exclusively to distant skin or subcutaneous sites (M1a) have a 1-year survival rate of 59%, while patients with metastases to any other visceral sites (M1b-M1c-M1d) have a 1-year survival rate of 41% [3]. Therefore the involvement of sole skin in the metastatic process delineates a better prognosis compared to the other most common metastatic sites, such as visceral or brain metastases [11]. On this matter, Niebling et al., evaluated the prognostic role of microsatellites in 69 cases of primary cutaneous melanoma with microsatellites, showing a 5-year melanoma-specific survival of 53%. Furthermore they demonstrated that microsatellites were correlated with a higher risk for sentinel node positivity and loco-regional recurrences. Therefore, they demonstrated that melanomas with microsatellites are aggressive tumors, showing a meaningfully worse disease-free survival, overall survival, and melanoma-specific survival [12]. Melanoma cells can metastasize to the skin both through lymphatic invasion or through direct spreading into the adjacent epidermis [3]. On this matter, Bastian et al., demonstrated that, in acral lentiginous melanoma, isolated melanocytes with amplifications (11q13 and 5p15) in the epidermis up to 3 mm beyond the histologically recognizable extent of the melanomas can be detected, thus revealing a mechanism of local dissemination beyond histologically detectable margins [13]. Furthermore, North et al., demonstrated that these melanoma cells extend significantly into normal skin without a correlation to tumor thickness or size [14]. Finally, Gambichler et al., demonstrated that, among 451 patients with melanoma with a negative sentinel lymph node, subtypes such as invasive lentigo maligna melanoma and acral melanoma were correlated with a higher risk of recurrence. This data confirmed the poor prognosis of acral melanoma and highlighted the need for more strict follow-up measures in patients with invasive lentigo maligna and acral melanoma, despite having a negative sentinel lymph node [15].

## 3. Pathogenesis

In normal conditions, melanocytes are linked to the keratinocytes in the basal layer through adhesion molecules such as transmembrane glycoprotein E-cadherin [16]. In melanoma, as in other cancers, one of the main mechanisms underlying cancer cell dissemination is the epithelial-to-mesenchymal transition (EMT), a multi-step process involving several molecular and cellular modifications, such as the downregulation of gene coding for epithelial proteins (i.e., E-cadherin/CDH1 and occludin/OCLN, etc.) and the upregulation of gene coding for mesenchymal markers (i.e., N-cadherin/CDH2, fibronectin/hFN1, and vimentin/VIM, etc.) [17,18]. These down- and up-regulations result in changes in the cellular cytoskeleton and adhesion molecules that facilitate cancer cell transition towards a metastatic variant. Since melanoma originates from neural crest-derived melanocytes and not from epithelial cells, it does not progress through classical EMT, but progresses through a distinct EMT-like process [19,20,21]. In particular, one of the main processes underlying the EMT in melanoma cells is the switch in the expression from E-cadherin to N-cadherin that has been demonstrated to be correlated with the loss or inactivity of PTEN, thus regulating the change in cadherin phenotype during melanoma progression [22]. Increased N-cadherin expression and decreased E-cadherin expression result in increased cell motility, enhancing the cell migratory capacity, increasing invasiveness, and downregulating apoptosis [23,24]. A loss of E-cadherin inhibits the melanocyte regulation by keratinocytes, while increased levels of N-cadherin seem to enable melanoma cells to migrate into the dermis, where melanoma cells interact with dermal fibroblasts and vascular endothelial cells that express the same cadherin. This series of events seems to have a key role in the metastatic spreading of melanoma out of its epidermal origin [25,26]. Another change that has been demonstrated as endorsing the metastatic features of melanoma cells is the upregulation of VE-cadherin. In fact, the expression of VE-cadherin on melanoma cells lets tumor cells adhere to endothelial cells, thus enabling blood vessel penetration [27]. Another crucial factor in melanoma metastasis to the skin is represented by the interactions between chemokines C-C motif chemokine receptor 10 (CCR10) and C-C motif chemokine ligand 27 (CCL27) [28]. CCL27, a chemokine expressed in the epidermis by normal keratinocytes, seems to interact with the chemokine receptor CCR10, which is expressed in melanoma cells [29]. In a mouse model, a blockage of CCL27 showed an inhibition of the development of skin metastasis [30]. As already mentioned, there are three different metastatic ways that the spreading of primary cutaneous melanoma occurs, such as satellite or ITM, lymph node metastases, or distant metastases [31]. The occurrence of ITM is almost unique to melanoma. The mechanisms underlying ITM have not been fully elucidated, but it seems to be related to the entrapment of tumor cells within the dermal and subdermal lymphatics between the site of the primary tumor and the local draining regional lymph nodes [32,33]. Other events that have been linked to the dissemination of melanoma cutaneous metastases are angiotropism and the “extravascular migratory metastasis” (EVMM). This latter refers to the capacity of melanoma and other tumors (such as glial tumors) to use the external surfaces of blood vessels as way to increase cell migration and the possible implantation of metastases [34]. Other possible mechanisms include tumor cell dissemination through tissue fluid and spread via the bloodstream [35,36]. Melanoma cells follow different paths of metastatic spread, with the primary tumor location and tumor thickness being the most frequent predictive factors of cutaneous dissemination. Indeed, tumors located in the extremities and trunk with a Breslow thickness (less than 0.76 mm and more than 1.5 mm, respectively) have a higher risk of developing satellite or ITM [3,37]. Another important factor influencing the different metastatic pathways has been demonstrated to be sex related. Mervic et al., demonstrated that, among 7338 patients with primary cutaneous melanoma, females displayed a significantly higher predilection for primary satellite or ITM; in particular, in 18.7% of men and 29.2% of women, the first metastasis was satellite or ITM [38]. Zaremba et al., in a study with 191 patients with initially unresectable stage III ITM, showed that patients with unresectable ITM without distant progression were more often female, older, and had a primary tumor on the leg [39].

## 4. Clinical and Dermoscopic Features

CMMs can arise on all anatomic sites, usually in the same anatomic area of the primary tumor [40]. They can appear as single or, more frequently, multiple dermal or subcutaneous nodules [41]. They usually present as firm, pigmented, reddish or skin-colored papules and nodules, sometimes ulcerated. Skin metastases are usually asymptomatic, although in advanced stages, they can present with ulceration, bleeding, and superinfection; furthermore, they can cause symptoms related to compression on nearby tissues. Histologically, they are characterized by gatherings of atypical melanocytes within the dermis with a well-defined Grenz zone and thinning of the epidermis, usually with a low or absent inflammatory infiltrate [40]. They also show lymphovascular permeation and positivity for HMB-45 [42]. Sometimes, the histological pattern can be indistinguishable from other benign lesions such as blue nevi or compound nevi [43]. Numerous clinical variants of skin metastases have been reported, mostly as rare presentations or case reports. Some of them can mimic primary cutaneous carcinomas such as keratoacanthoma, squamous cell carcinoma, or basal cell carcinoma [42]. Some authors have also described various clinical manifestations, such as erythematous patches or plaques [44], erysipelas-like lesions [45], telangiectatic papulovesicles, purpuric plaques mimicking vasculitis [46], and alopecia aerate-like scalp lesions [47,48] (Table 1).

Since CMMs may sometimes represent the first clinical sign of melanoma, it is important for clinicians to recognize their dermoscopic patterns. In fact, dermoscopy has been demonstrated to represent a useful tool that can help physicians with the early identification and differentiation of CMMs from other benign and malignant skin lesions. To date, many authors have described various dermoscopic features of the cutaneous metastases of melanoma, but no precise and standardized dermoscopic patterns have been identified. Rubegni et al., described five different dermoscopic patterns: a homogeneous pattern characterized by diffuse pigmentation (red, brown, gray, or grayish black) without any other definite structures; a saccular pattern resulting from oval junctional nests containing atypical melanocytic cells; an amelanotic one, identified by dermal localization and a lack of pigment; a vascular pattern, which is considered to be the most frequent in CMM and characterized by punctate vessels in thin lesions and corkscrew vessels in thick ones; and finally, a polymorphic pattern, characterized by chaotic structures and vessels [9] (Table 2). On the contrary, Costa et al., described five dermoscopic patterns of CMM (blue nevus-like, angioma-like, nevus-like, vascular, and unspecific) that demonstrated a good inter-observer agreement, as well as a good sensitivity and specificity [48].

Finally, Kostaki et al., demonstrated the association between features and clinicopathological melanoma characteristics, showing that vascular structures were more frequent in the metastases of deeper tumors, while nevus-like structures were more common in the metastases of thinner tumors [49]. Another crucial aspect of CMM is the differential diagnosis with skin metastasis derived from other tumors. Breast cancer is a tumor with a higher risk of cutaneous involvement in women, followed by lung, gynaecological malignancy, head and neck (including the thyroid), and gastrointestinal tract, while in men, the most common primary site is the gastrointestinal tract, followed by lung, head and neck (including thyroid), and urinary system [50].

## 5. Treatment

Identifying the appropriate therapeutic strategies for the treatment of CMMs has always been challenging due to their resistance to standardized approaches and frequent relapses. Considering the high frequency of local relapse, the goal of both systemic and local therapy is a treatment that can guarantee R0 with a relatively minimal invasive approach. The choice of the most appropriate treatment depends not only on the site of metastases location and the extent of the disease or the patient’s conditions, but is also influenced by the availability of the different techniques according to different institutions and countries. Treatments vary from simple surgery resections to topical or intralesional local injections, or a combination of these techniques with the most recent systemic immune or target therapies [51] (Table 3).

### 5.1. Surgery

The first-line treatment for ITM or distant metastasis with no evidence of distant metastatic disease to other sites is represented by surgical resection. Usually, surgery is reserved for patients with a limited number of lesions, although it can also be applied to multifocal metastases in palliative settings [52]. In patients with in-transit or satellite metastases restrained to the skin and subcutaneous tissue, the most applicable treatment is complete excision with a small margin. Although most patients usually experience widespread metastatic disease, a complete resection of the metastatic disease is associated with prolonged survival in up to 40% of cases [53]. In some selected patients, laser ablation should be considered. Kandamany et al., performed carbon dioxide ablation on 16 patients with in-transit cutaneous malignant melanoma metastases that had previously undergone surgical procedures for disease control. Among them, ten patients achieved complete remission at 1-year follow up [54]. Although according to the data in the literature, recurrence at treatment sites remains extremely low, the overall data of the outcomes of CO_2_ laser ablation clearly need much more research and clarification [55].

### 5.2. Radiation Therapy

Radiation therapy (RT) has been widely used in palliative settings for both skin and subcutaneous metastases, following the concept that melanoma is resistant to radiation [56,57]. This hypothesis has been definitely reverted, demonstrating that some melanomas show a high sensitivity to radiation [58,59]. Habermalz et al., reported a response rate of >90% in patients with CMMs treated with fractions of 6 Gy and higher, although there was no response in patients irradiated with less than 6 Gy [60]. On the other hand, Overgaard et al., described a CR of 66% and PR or 33% in 35 lesions of 14 patients with CMMs, showing no difference between a high or low total dose of RT [61].

Moreover, in a retrospective analysis, Fenig et al., showed a 52% response rate in 69 metastatic patients who received radiotherapy in a palliative setting [62]. Finally, Olivier et al., demonstrated a partial response in 86 (75%) of 114 lesions in 84 patients with CMMs. In this study, patients treated with >30 Gy had a considerably longer freedom from disease progression compared to patients given ≤ 30 Gy [63]. The most frequent adverse events reported in the above mentioned studies were erythema and fibrosis [61,62,63].

### 5.3. Isolated Limb Perfusion

Isolated limb perfusion (ILP) is a technique that was first introduced in 1958 by Creech and Krementz, which consists of the administration of chemotherapy agents exclusively to the involved limb, isolated from the systemic circulation [64]. Chemotherapy drugs are administrated throughout a canulated artery and vein using an extracorporeal bypass circuit, therefore permitting a much higher administration of drugs solely to the isolated limb with a concentration that would not be tolerated systemically [65,66]. Authors have reported overall response rates of 65–100% and complete response rates of up to 65% [67]. The alkylating agent melphalan alone is the most common treatment option for ILP [68]. Many authors have evaluated the efficacy of single-agent melphalan, demonstrating a median OR of 84.5% (range 79–90.6%) with a median CR of 57% (range 33–64%) [66]. Another treatment option for ILP is a combination of melphalan plus tumor necrosis factor (TNF-α). TNF-α is a cytokine with antitumor effects probably mediated by a specific action against tumor blood vessels, thus enhancing the cytotoxic effect of melphalan [69]. In previous studies, it has been demonstrated that melphalan plus TNF-α ILP has higher overall and complete response rates than melphalan alone, with a median OR of 93% (range 67–100%) and a median CR of 61.5% (range 33–76%) [70,71,72]. An additional combination therapy reported in the literature is melphalan plus D-actinomycin. In two studies with 243 patients who underwent ILP with the combination of melphalan with D-actinomycin, the authors demonstrated an OR of 87.65% (range 80.70–96.60%) and a CR of 76% (range 62.8–89.20%) [73,74]. In one study, the authors analyzed the role of melphalan plus dacarbazine in 100 patients who underwent ILP for ITM, reporting an OR of 90.80% and a CR of 73.40% [75]. Regarding progression-free survival and overall survival, there were no differences between single-agent melphalan and combination therapies [76,77,78]. Toxicity can be divided into loco-regional or systemic and acute or long-term. According to Wieberdink et al., acute reactions to ILP can be classified as follows: I, no reaction; II, slight erythema or edema; III, considerable erythema or edema with some blistering, slightly disturbed motility permissible; IV, extensive epidermolysis or obvious damage to the deep tissues causing a definite functional disturbance and threatening or manifesting compartmental syndrome; and V, a reaction that may necessitate amputation [79]. The most frequent long-term reaction is lymphedema, followed by muscle atrophy or fibrosis, limb malfunction, neuropathy, pain, and recurrent infection [80].

### 5.4. Isolated Limb Infusion

Isolated limb infusion (ILI) is a minimally invasive technique developed in 1990 by Thompson et al., that allows for the administration of high-dose melphalan ± actinomycin-D to the extremities of patients affected by locally advanced or ITM [81]. The alkylating agent melphalan induces a pro-inflammatory microenvironment inhibiting regulatory T cells; it also directly inhibits the DNA replication of melanoma cells [82]. On the other hand, actinomycin-D is an antineoplastic antibiotic that inhibits the transcription of DNA by RNA polymerase and controls topoisomerase II activity. The combination of melphalan and actinomycin-D is thought to enhance response rates with low toxicity rates [83,84]. Compared to ILP, ILI has a much lower morbidity and minor systemic toxicity. Therefore, it may be the best option for treating elderly and frail patients who cannot tolerate other accessible treatments, and it can also be repeated after disease recurrences [85]. Different from ILP, in ILI, arterial and venous catheters are placed percutaneously, in order to administer the cytotoxic agents to the affected limb [86]. The cytotoxic infusate is not oxygenated, enhancing, through the hypoxia and acidosis of the limb, the anti-tumor activity of the cytotoxic substances [87]. O’ Donoghue et al., reported an ORR of 59% in 163 patients with ITM, also demonstrating a significant difference (*p* = 0.04) between upper (76.9%) and lower extremities (55.1%) [88]. Teras et al., analyzed the safety and efficacy of ILI in 160 octogenarian and nonagenarian patients, assessing similar responses and regional control rates compared to those of younger patients, but with a shorter overall and melanoma-specific survival [89]. Muilenburg et al., analyzed different response rates according to the tumor burden (low or high) in 160 patients with ITM. Patients with a low tumor burden had an overall response rate (ORR) of 73% with a 50% CR, compared to an ORR of 47% with a 24% CR in patients with a high tumor burden. Patients with a low disease burden had an increased median PFS of 6.9 versus 3.8 months and an increased median OS of 38.4 versus 30.9 months [90]. In recent years, the widespread prevalence of systemic targeted and immunotherapy has allowed for the association of systemic immunotherapy combined with local chemotherapy through ILI, in order to increase the responses in locoregional melanoma. On this matter, Carr et al., compared the outcomes of melanoma patients treated with ILI in the United States of America (USA) and Australia (AUS), demonstrating that, in the US, patients who received ILI after ipilimumab approval in 2011 had a significantly improved OS [81].

### 5.5. Electrochemotherapy

Electrochemotherapy (ECT) is a treatment that combines electroporation (EP), a mechanism that allows the cell membrane to become permeable through the application of electrical pulses, and the administration of chemotherapeutic agents [91]. The two most common chemotherapy agents used in ECT are bleomycin and cisplatin, that, as widely demonstrated, exert their cytotoxic activity thorough damage to nuclear DNA [92]. Both these drugs represent the best choices for ECT, since they both disperse scantily throughout the cell membrane and are highly cytotoxic inside cells [93]. ECT was first approved for head and neck squamous cell carcinomas following a clinical trial in 1993, but nowadays, it finds its main applications in melanoma and Kaposi sarcoma [94,95]. Kunte et al., demonstrated that, in 151 patients with metastatic melanoma, among the 394 lesions treated, 306 (78%) showed OR, while 229 (58%) showed a complete response [96]. According to the European expert consensus, the ideal patient with in-transit melanoma metastases that could benefit from ECT should have fewer than 10 tumors smaller than 3 cm, or fewer than 20 smaller than 1 cm, and they should involve the same limb segment over an area not exceeding 10 cm [94]. ECT can induce immunogenic cell death (ICD), stimulating the immune system, mainly dendritic cells (DCs) and macrophages, through damage-associated molecular patterns (DAMPs) such as calreticulin (CRT), heat shock proteins, high-mobility group box 1 (HMGB1), type I IFN, and ATP [97,98]. Furthermore, it has been demonstrated that T cells play a key role in the immune response for the local and systemic effects of ECT [99,100,101]. Roux et al., demonstrated that ECT followed by TLR-9 ligands, CpG oligodeoxynucleotides (CpG ODN), in three murine tumor models synergized and enhanced the local effect and systemic T-cell-dependent antitumor activity [100]. Furthermore, it has also been demonstrated that ECT can modify the DC and T cell populations in the lesions of melanoma patients who have undergone bleomycin ECT. The latter induces the migration of Langerhans cells to the draining lymph nodes, followed by the recruitment of plasmacytoid DCs (pDCs) and dermal CD1 DCs (dDCs) to the treatment site [102]. On the other hand, Di Gennaro et al., also demonstrated that ECT significantly reduces the percentage of T regulatory (Treg) cells [103]. Besides pre-clinical observations, a strong clinical rationale for the association of ECT with immunotherapy has been provided by several retrospective analyses and case reports. In two reports, assessing the safety and efficacy of ECT combined with the CTLA-4 inhibitor ipilimumab in, respectively, 15 and 28 patients with advanced melanoma, a local ORR of 67% was reported, while the systemic ORR was only 19%. The systemic ORR significantly increased with a different combination, namely ECT plus PD-1 inhibitors (pembrolizumab or nivolumab), which induced a systemic ORR of 40% in five patients with metastatic melanoma [104,105]. Pain and erythema were the most common adverse events, followed by necrosis, infection, ulcerations, muscle spasms, and nausea [106,107].

### 5.6. Intralesional Therapies

In recent years, many authors have demonstrated the efficacy of intralesional immunotherapies for the treatment of cutaneous and subcutaneous melanoma metastases, such as PV-10 Rose Bengal, Bacillus Calmette Guerin (BCG), interleukin-2 (IL-2), interferon alpha, non-oncolytic viral therapies (Toll-like receptor agonists), and oncolytic viral therapies (T-VEC, CAVATAK, and HF10) [108,109,110]. In a phase II trial, Radny et al. evaluated the role of intralesional IL-2 in a total of 245 CMMs in 24 patients with stage III or IV melanoma. With single doses varied from 0.6 to 6 × 10^6^ IU, a CR was seen in 209 lesions (85%) and a PR in 21 (6%), with limited toxicity, suggesting a possible role of IL-2 in the treatment of CMM [111]. Furthermore, few case reports have analyzed the role of interferon-β. In particular, Takahara et al., demonstrated how intralesional IFN-β therapy could be an option for improving the quality of life of patients with ITMs refractory to ICIs [112].

#### T-VEC

Among intralesional therapies, T-VEC represents an innovative immunotherapy approach in cancer treatment [109]. It is the first engineered oncolytic herpes simplex virus type 1 (HSV-1) that is intra-lesionally administered and duplicates in the tumor mass causing the lysis of tumor cells, presenting tumor-derived antigens to CD8+ T-cells and thus enhancing anti-tumor immunity [113,114,115]. Following the Phase III OPTIM study with 436 patients with locally advanced or metastatic melanoma, T-VEC has been approved in the United States, Australia, UK, Germany, Switzerland, and France [116]. Louie et al., showed an ORR of 57% in 80 patients with stage IIIB–IV melanoma after 9 months of T-VEC, with mild adverse events occurring in 22% of patients [117]. Franke et al., demonstrated an ORR of 88.5% in 26 patients with stage IIIB–IVM1a melanoma. In particular, 16 (61.5%) patients showed a CR and 7 (26.9%) reached a PR [118]. Finally, Perez et al., analyzed the safety and efficacy of T-VEC in 27 patients with locally advanced melanoma, showing an ORR of 56.5% [119]. One crucial and promising aspect of T-VEC is that its efficacy is not confined only to injected lesions, but extends also through an abscopal effect on metastasis in distant sites [120]. The toxicity of T-VEC is usually mild and tolerable, with the most common symptoms being edema and erythema at injection sites, chills, fever, and fatigue, thus representing a safe and valid new approach for melanoma treatment [110,121].

### 5.7. Topical Therapies

#### 5.7.1. Imiquimod

Imiquimod is a Toll-like receptor (TLR) 7 and 8 agonist that exerts its activity by stimulating dendritic cells, activating the Th1-prevalent cellular immune response, and stimulating the activity of tumor-specific cytotoxic CD8+ T cells [122,123]. Imiquimod has been approved by the Food and Drug Administration (FDA) for the treatment of genital warts, actinic keratoses, and basal cell carcinomas [124]. To date, the data of imiquimod for the management of CMMs are restricted to case reports and clinical trials. Green et al., reported the results of a phase I/II trial investigating the role of topical imiquimod applied daily for 4 weeks and intralesional IL-2 in 13 patients with ITM refractory to previous treatments. Among 182 lesions, the authors reported a CR in 74 lesions (40.7%) with a good safety profile [125].

In a retrospective study, Shi et al., evaluated the efficacy of intralesional IL-2 associated with topical imiquimod and retinoids in 11 patients with CMMs, showing a complete response in all 11 patients at 24-month follow-up, thus representing a valid alternative to surgery in the treatment of CMMs [126]. In a case series of 20 patients with CMM treated by a combination of cryotherapy and imiquimod, Rivas-Tolosa et al., reported a CR in eight (40%) patients and a PR in five (25%) patients [127]. Finally, in a phase II trial, Teulings et al., analyzed the role of a combination of imiquimod plus monobenzone, a skin-bleaching agent that can induce vitiligo. Among 21 patients treated for 12 weeks with the association of imiquimod and monobenzone, there was a local regression of cutaneous metastases in 38% of patients that increased to 52% after prolonged therapy was reported [128]. Following the evidence of the above-mentioned studies, imiquimod in association with other drugs can be considered to be a valid option for the treatment of CMM, and also alone in a palliative setting [129].

#### 5.7.2. 5-Fluorouracil

5-Fluorouracil is an anti-metabolite that induces the apoptosis of cancer cells through the inhibition of DNA synthesis. Topical 5-fluorouracil has been approved for the treatment of Bowen’s disease and actinic keratosis [130]. Topical 5-FU seems to be more efficient when applied in combination with another treatment such as imiquimod. In fact, Florin et al., reported the results of topical application, 5 days per week, of 5-fluorouracil in the morning and imiquimod at night. Among the 45 lesions treated, 19 reached a CR, while 25 lesions underwent a PR, and the treatment was well tolerated [131].

#### 5.7.3. Diphencyprone

The mechanism of diphencyprone is still unclear, but it is a contact sensitizer that, to date, has been used mainly as an immunotherapy for warts and alopecia areata, although it has not been approved by either the Food and Drug Administration or the European Medicines Agency [132]. Gibbons et al., reported data on 16 patients with CMMs treated with topical diphencyprone, describing a 37.5% complete response and a 25% partial response. The treatment was well tolerated [133]. Among 50 patients with CMMs, Damian et al., reported a complete clearance of cutaneous disease in 46% and a partial response in 38% of patients, assessing that diphencyprone should be considered for patients with skin metastases refractory to other therapies [134]. Furthermore, the role of diphencyprone in association with systemic immunotherapy, such as pembrolizumab and nivolumab, has been evaluated, with promising results [135,136].

### 5.8. Systemic Immunotherapies

As widely demonstrated, in the last decade, the use of targeted therapies and immunotherapies has revolutionized the treatment and prognosis of melanoma. Since pilot studies of immunotherapy for metastatic melanoma have not comprised subgroups of ITM for analysis, data on the efficacy of these drugs for the treatment of ITM are scarce. Therefore, Tie et al., retrospectively analyzed 54 patients with ITM (stage III) treated with immunotherapy, with a median follow up of 15 months; in particular, forty patients (74%) were treated with PD-1 inhibitor (pembrolizumab or nivolumab), eight (15%) with anti-CTLA-4 (ipilimumab), five (9%) with combination anti-PD-1/anti-CTLA-4 (ipilimumab and nivolumab or pembrolizumab), and one (2%) with a combination of anti-PDL-1 (atezolizumab) and MEK inhibitor (cobimetinib). The ORRs were 58% for single-agent anti-PD-1, 38% for single-agent anti-CTLA4, and 40% for anti-PD-1/anti-CTLA-4 [137]. Holmberg et al., in a multicenter retrospective study, evaluated, in 287 patients with ITM, the efficacy of the PD-1 inhibitor (nivolumab or pembrolizumab) and/or CTLA-4 inhibitor (ipilimumab) according to the RECIST criteria, with an overall response rate of 56% [138]. These data confirm the efficacy of immunotherapies also in the treatment of CMMs, and open up a new scenario of combinations with already approved local therapies such as surgery and intralesional therapies.

## 6. Conclusions

The lack of consolidated data and randomized trials on the safety and efficacy of treatment exclusively for ITM is probably at the base of the variable outcomes reported for the different treatments in case reports and case series. It has not yet been clarified why, in many patients, these tumors spread rapidly to regional nodes or distant sites, while, in other patients, they remain confined within a single loco-regional area for months or years. Therefore, new research and studies should focus on the pathogenesis and molecular mechanisms of ITM in order to shed light on the mechanisms underlying the different behavior of ITM in different patients. Despite the new immune local and systemic therapies dramatically changing the prognosis of metastatic melanoma, the management of in-transit and distant cutaneous metastases still remains an unmet need. Local treatments such as surgery, intralesional, topical, or locoregional treatment guarantee control of the disease, but rarely have a curative impact. Therefore, a randomized trial of a new combination of the already available local and systemic treatments is required in order to identify the right treatment for a disease that has a very high impact on the quality of life of patients.

## Figures and Tables

**Table 1:** Most common clinical manifestations of CMM.

Keratoacanthoma-like [42] (crateriform ulcerated papule or nodule)
Squamous cell carcinoma-like [42] (hyperkeratotic-scaly papule or nodule)
Basal cell carcinoma [42] (pearly papule or nodule with arborizing telangectasias)
Erythematous patches or plaques [44]
Erysipelas-like lesions [45] (marginated erythematous plaque with ridge-like borders)
Alopecia aerate-like scalp lesions [47,48] (circular area of alopecia)
Vasculitis-like [42] (purpuric macules or papules)

**Table 2:** Most common dermoscopic patterns of CMM [9].

Homogeneous pattern	Diffuse pigmentation red, brown, gray, or grayish black
Saccular pattern	Oval junctional nests containing atypical melanocytic cells
Amelanotic pattern	Dermal localization and lack of pigment
Vascular pattern	Punctate vessels in thin lesions and corkscrew vessels in thick ones
Polymorphic pattern	Chaotic structures and vessels

**Table 3:** Currently available treatment options for CMM.

Regional	Surgery [52] Radiation therapy [52] Isolated limb perfusion (melphalan ± actinomycin-d, tnf-α, dacarbazine) [52] Isolated limb infusion (melphalan ± actinomycin-d) [52] Electrochemotherapy (bleomycin and cisplatin) [52]
Intralesional	T-vec [52]
Topical	Imiquimod [52] 5-fluorouracil [52] Diphencyprone [52]
Systemic Immunotherapies	Pembrolizumab (anti PD-1) Nivolumab (anti PD-1) Ipilimumab + Nivolumab (anti CTLA-4 + anti PD-1) [52]
